# Chloride of Lime in Snake Bite

**Published:** 1895-08

**Authors:** 


CHLORIDE OF LIME IN SNAKE-BITE.—Drs. Phisalix and Bertrand (*Medical. Week*, 1895, IV., p. 307).—Various substances have been suggested as antidotes of snake poison—chlorine, bromine, iodine, and various combinations of these, such as trichloride of iodine and the hypochlorites. The latter, especially have been recommended by Dr. Calmette, who claims that in addition to their power of counteracting snake poison, these substances possess the property of producing the same vaccinal reaction as heated venom. The authors have carried out a series of experiments with a solution of chloride of lime (calcium hypochlorite) the results of which seem to show that this substance protects the organism against the bite of venomous snakes—not, as Dr. Calmette assumes, by determining the formation of an anti-toxic substance or by entering the circulation and there destroying the venom, as it does in a test tube, but purely by its local action.

It destroys the poison and mortifies the tissues, thus interfering with the absorption of the poisonous matter.

It follows, therefore, as a practical application of this fact that injections of chloride of lime at another point than that of the bite have no immunizing effect, and are consequently useless. If this antidote be employed, it should be injected deeply under the skin at the very spot where the fangs of the snake have entered.

